# Cultural Differences in The Content of Employees’ Psychological Contract: A Qualitative Study Comparing Belgium and China

**DOI:** 10.5334/pb.498

**Published:** 2020-06-01

**Authors:** Jiahong Du, Tim Vantilborgh

**Affiliations:** 1 Department of Work and Organizational Psychology, Vrije Universiteit Brussel, BE

**Keywords:** psychological contract, mutual obligations, culture, qualitative, social exchange

## Abstract

This study qualitatively explores differences in psychological contract (PC) content between Belgian and Chinese employees, while attempting to understand these differences from the perspective of cultural values. We build on theory concerning horizontal and vertical individualism/collectivism to interpret differences in PC content. 21 Chinese and 20 Belgian employees were interviewed, with results indicating that Belgian interviewees’ PCs tend to be balanced with an emphasis on egalitarian interpersonal relationships, reflecting a horizontal collectivist culture. We propose that a “culture of compromise” forms a fitting description for Belgian interviewees’ PCs. For Chinese interviewees, the PC was characterized by mixed contracts aligning with the ideology of ‘Utilitarianistic Guanxi’, which forms a Chinese philosophy that combines the pursuit of profit with objective goods, reflecting a culture of vertical collectivism.

The psychological contract (PC) captures an employee’s beliefs about mutual obligations between her- or himself and the employer (Rousseau & Tijoriwala, 1998) and is a key construct to explain employee attitudes and behaviors (Zhao, Wayne, Glibkowski, & Bravo, 2007). Scholars have noted that the majority of the PC research uses Western samples and overlooks that PCs can differ substantially between cultures (Thomas, Au, & Ravlin, 2003). The few PC studies that compared countries focused on Western (e.g., US) and South Asian countries (Zhao et al., 2007). Scholars have also started to compare China and Western countries, given the rise of China in the global economy. However, most of these studies centred on South China (e.g., Hong Kong), which does not represent the entire cultural heritage of China, resulting in a lack of research focusing on North and Central China (Ravlin, Liao, Morrell, Au, & Thomas, 2012).

Given that organizations increasingly operate in a globalized context with employees from various cultural backgrounds, it is essential to gain a better understanding of how culture shapes PCs. We aim to qualitatively explore differences in the PC of employees from two distinct cultures, focusing on Belgian and Chinese employees, who have been shown to differ on several cultural values (Hofstede, 2001). Employment relationships and PCs in Belgium are similar to those in other Western-European countries (Psycones, 2006), yet are quite distinct from those in Eastern countries like China (Yang et al., 2012). Our study thus fits into a broader line of research that compares psychological phenomena in a Belgian and Chinese context (e.g., Wuyts, Chen, Vansteenkiste, & Soenens, 2015). We use this comparison of Belgium and China as a case to illustrate the subtle, yet important, nuances that can emerge in the perceptions of mutual obligations that form an employee’s PC.

Our study contributes to the literature in two major ways. First, we illustrate that current conceptualizations of PCs, which are based predominantly on Western research, may not accurately capture the content of Chinese employees’ PCs. This illustration has ramifications for both scholars and practitioners. For the former group, acknowledging the cultural differences in PCs can help guide future research, for example by laying the groundwork for the development of culture-specific measures of PC content. For the latter group, realizing how cultural differences manifest themselves in the PC of employees can help to manage culturally diverse teams at work. Overall, we address calls to examine the effect of different cultural values in forming individuals’ PCs (Shen, Schaubroeck, Zhao, & Wu, 2019). Second, our qualitative approach allows us to explore the underlying causes of cultural differences in PCs. We integrate research on horizontal and vertical individualism/collectivism with the PC literature, to advance our theoretical understanding of how culture shapes PCs. In the following sections, we introduce the literature on PCs and cultural differences, highlighting how cultural values can help us to understand cultural differences in PC content.

## Psychological contracts

The PC captures an individual’s beliefs of a reciprocal exchange between two parties (Rousseau, 1989). This implies that a promise has been made (implicitly or explicitly) and a consideration offered in exchanged (Rousseau, 1989). However, perceptions of mutual obligations can also form in the absence of promises, as PCs can also be based on general expectations (Montes & Zweig, 2009). Such expectations often capture more normative beliefs about exchange relationships, such as societal norms, or they can be based on an employee’s prior work experiences (Rousseau, Hansen, & Tomprou, 2018). Overall, the PC forms a powerful determinant of employee behaviour (Hansen & Griep, 2016).

MacNeil (1985) argued that two PC types can be distinguished: *transactional PCs* involve well-defined obligations with a specific time frame, tangible resources, and all the terms in the exchange are clear and explicit; *relational PCs* involve a mentoring relationship and intangible socio-emotional resources, an unclear and open-ended time-frame, and terms that are highly subjective, implicit, and amorphous (Rousseau & Tijoriwala, 1998). Transactional and relational PCs have different functions for individuals and organizations. Transactional contracts can serve as a trial run before a long-term relationship, such as probationary employment (Rousseau & McLean Parks, 1993). Relational contracts serve a broad array of needs and therefore lead to higher individual performance and labour productivity (Rousseau & McLean Parks, 1993). Thus, transactional contracts can be seen as egoistic or instrumental models, whereas relational contracts rely on a collectivistic or socialized model (Rousseau & McLean Parks, 1993). While transactional contracts can attract employees temporarily, relational contracts are better to establish a long-term relationship.

In addition, Rousseau (2000) identified a *balanced PC* type, which relates to a combination of an open-ended relational agreement with the transactional feature of performance-based rewards. Balanced contracts include three components, namely external employability, internal advancement, and dynamic performance (Rousseau, 2000). It is characterized by a longer time-frame and flexibility of employment agreements to accommodate changing environments (Dabos & Rousseau, 2004). Another more recent addition to existing PC classifications is the *ideological PCs*, which contain credible commitments to pursue a valued cause or principle that are implicitly exchanged within the employment relationship. It might entail taking initiative to serve the needs of the organization’s clients, acting as an advocate for the organization’s espoused cause, or sacrificing non-work time for the organization’s ideological mission (Thompson & Bunderson, 2003).

Coinciding with increasing globalization, there is a growing interest in the PC in different countries. So far, this interest translated into distinct results in different countries. Chinese scholars—who have been studying PCs for several decades—proposed new PC typologies, yet these typologies share many resemblances with the abovementioned Western typologies (e.g., Chen, Tsui, & Zhong, 2008; Hui, Lee, & Rousseau, 2004). One goal of our study will be to reconcile findings from both streams of research and to qualitatively explore cross-cultural differences in the content of employees’ PCs. Most studies on cultural differences in PCs used a quantitative approach (e.g., Psycones, 2006). However, qualitative studies may offer a richer understanding of subtle cultural differences in PC content, because they build on the participants’ personal experiences (e.g., Thomas et al., 2010). Given that the content of the PC is highly subjective, we believe that an exploratory qualitative approach into these cultural differences is warranted.

## Culture and psychological contracts

Various definitions of culture can be found in the literature. Hofstede (1980) defined culture as the “*collective mental programming*” that is shared by people who were conditioned by the same education and life experiences. Thomas and colleagues (2003) described culture as systems of values, attitudes, beliefs, and behavioral meanings shared by members of a social group or society and learned from previous generations. When we zoom in on the role of culture, we can see that culture influences how individuals perceive and process information within their environment (Coyle-shapiro, Pereira Costa, Wiebke, & Chang, 2019; Kickul, Lester, & Belgio, 2004; Thomas et al., 2003). According to Rousseau and Schalk (2000), culture influences employees’ PCs in three ways: the extent to which employees can negotiate the terms of their PC; the extent to which employees perceive promises as binding; and the extent to which obligations create differences between in- and out-groups. Thomas and colleagues (2003) argued that culture influences employees’ PCs through motivational and cognitive mechanisms. Cultural values influence how employees cognitively organize and process information related to the exchange relationship with their employer, and they shape the motives or needs of employees which thus determines what they consider important obligations in the exchange relationship. This motivational pathway can be used to explain why cultural differences may translate into differences in PC content. Employees tend to seek out PCs that allow them to attain goals that they find valuable (Rousseau et al., 2018). When employees from distinct cultures are motivated by different goals or needs, then this should translate into differences in PC content.

Building on the idea that cultural values may be related to PC content, McLean Parks and Smith (1998) proposed an alternative PC typology. Their PC typology maps onto the horizontal versus vertical orientations that have been identified within the individualism/collectivism cultural value (Singelis, Triandis, Bhawuk, and Gelfand, 1995). Based on this model, a map of four cultural profiles can be formed. Vertical collectivists regard themselves as an in-group but perceive members of the in-group to be different in terms of status. Authority and sacrifice characterize this type. Horizontal individualists are autonomous and independent, emphasising equality and uniqueness. Vertical individualists also have an independent self-concept, but accept differences in status, resulting in people competing to be the best. Horizontal collectivists have an interdependent self-concept, and consider in-group members to be generally equal (Thomas et al., 2010; Triandis, 2001). When linking the four PC types identified by McLean Parks & Smith (1998) to horizontal and vertical individualism/collectivism, *Instrumental* contracts can be related to horizontal individualism (e.g., Canada) and tend to be transactional with symmetric power; *Exploitive* contracts can be linked to vertical-individualism (e.g., France) and tend to be transactional with asymmetric power; Communitarian contracts link with horizontal collectivism (e.g., Norway) and tend to relational with symmetric power; while Custodial contracts relate to vertical collectivism (e.g., China) and tend to be relational with asymmetric power (Thomas et al., 2010).

In view of our study—in which we compare Belgian to Chinese employees—studies show that Belgians emphasize the importance of both egalitarianism and individualism (Boiger, De Deyne, & Mesquita, 2013; Psycones, 2006). Turning to China, a strong hierarchy is emphasized within an interdependent collective, as people emphasize unity within the in-group while closely following the instructions of their superiors (Hui et al., 2004; Thomas et al., 2010). These observations suggest that Belgians endorse a horizontal individualist culture, while Chinese endorse a vertical collectivist culture. Combined with McLean Parks & Smith’s (1998) research, this would imply that Belgians are characterized by transactional PCs with symmetric power, whereas Chinese are characterized by relational PCs with asymmetric power (Schalk & Soeters, 2008).

However, we believe that simply characterizing Belgian and Chinese employees as having transactional and relational PC types may be overly simplistic. First, we already mentioned that other PC types have been identified in more recent years, including balanced and ideological PCs (Vantilborgh et al., 2012). Taking these PC types into account may offer a more nuanced perspective on Belgian and Chinese employees’ PCs. Second, even when employees from different cultural regions would be described by similar PC types, they may still differ in the specific obligations that compose these PC types (Rousseau, Schalk, Schalk, & Schalk, 2000). For example, employees from two groups may be said to both have a relational PC, yet the first group could emphasize job security whereas the second group could emphasize self-development. Third, one should take into account regional differences within both Belgium and China. For example, Belgium contains three federal regions: Flanders, Wallonia, and Brussels, with 60% of the population living in Flanders (Toharudin, 2010). People in Flanders identify more with an Anglo-Saxon culture that emphasizes equality, creativity, and independence; whereas Wallonia is typically described as being more socialist and Brussels as being more liberal (Billiet, Maddens, & Frognier, 2006). Likewise, differences exist within China, as southern regions in China have been influenced more by Western culture (Fan, 2000). For example, people in mainland China (e.g., mandarins) focus on *Wuwei* (doing nothing and letting things take their own course, which is inspired by Taoism) and values such as eremitism (individualism value orientation). These values are less relevant when doing research in South China (Fan, 2000). Based on these considerations, we believe that an exploratory qualitative approach to studying cultural differences in PC content is warranted.

## Research questions

Our central research question is whether there are differences in PC types and specific PC content between Belgian and Chinese employees. Moreover, we will try to relate potential differences between PCs to the cultural values expressed by interviewees.

Our research questions concern the PC type that both countries’ respondents report, including transactional, relational, balanced, and ideological contracts. From previous studies, Westerners (including Belgians) tend to be individualistic and mainly attracted by explicit inducements (Schalk & Soeters, 2008), which relates to transactional contracts. However, research indicates that Belgian culture is intermediate between East Asian and West European cultures, with Belgians emphasising their own rights within the context of an interdependent social network (Boiger et al., 2013; Nisbett, 2010). Therefore, Belgian employees might report both elements of transactional and relational PCs, resulting in balanced contracts. Given these different perspectives on Belgian employees’ PCs, we set out to explore the following research question:

Research question 1: What types of PCs do Belgian employees report, and are these PC types related to cultural values (in particular, horizontal-vertical individualism/collectivism)?

For Chinese employees, several studies show that Chinese culture is characterized by a collective orientation, in which its members prioritize the group over individual goals, accept strict hierarchy and group control, and are strongly influenced by social rules (Nisbett, 2010; Shen et al., 2019; Triandis, 2001). As a result, Chinese employees’ PCs tend to be relational rather than transactional (Hui et al., 2004; Nisbett, 2010; Triandis, 2001). However, Chinese people who were born after the Cultural Revolution might value security more, such as financial stability (Wuyts et al., 2015). Moreover, the reciprocity principle is a critical element in Chinese culture (Westwood, Sparrow, & Leung, 2001), which is an essential component of “Guanxi” circles that are social connections that lend special favours to each other (Lee & Dawes, 2005). In a guanxi circle, behaviours are based on mutual reciprocity with implicit and explicit—or transactional and relational—benefits. These guanxi circles therefore might relate to both relational and transactional contracts meaning that both PC types might be reported by Chinese employees.

Research question 2: What types of PCs do Chinese employees report, and are these PC types related to cultural values (in particular, horizontal-vertical individualism/collectivism)?

Besides the exploration of the PC types and contents within two countries, respectively, we also address our central question based on cultural differences.

Research question 3: What differences of PC types do Belgian and Chinese employees have based on their cultural values?

## Method

### Sample

Two convenience samples (Chinese employees: *N* = 21; Belgian employees: *N* = 20) were interviewed. Chinese interviewees originated from the capital Beijing, the provinces of Inner Mongolia, Shandong, and Hebei, which are all from the north and central areas of China, while Belgian interviewees originated from the Flemish region, except for two interviewees who lived in Brussels. Interviewees were purposively sampled to obtain a match between Belgian and Chinese interviewees’ work environments and job characteristics and to improve comparability between the two samples (Yang et al., 2012). To gain a better understanding of cultural differences between our samples, we asked interviewees to fill out a survey that contained the Vertical and Horizontal Individualism and Collectivism scale (Singelis et al., 1995). This scale contains 32 items and measures horizontal individualism, horizontal collectivism, vertical individualism, and vertical collectivism. The reliability of the four scales was satisfactory (α_V-I_ = .74, α_H-I_ = .76, α_V-C_ = .72, α_H-C_ = .87). While we report how both samples score on this measure in Table 1, we do not rely on these data to empirically examine cultural differences. Rather, the qualitative data from the interviews forms the basis for our analysis of cultural differences in PC content. Apart from three older employees, all interviewees were between 25 and 40 years old. Chinese participants were on average 30.7 years old (*SD* = 5.95) and well educated (highest attained degrees: higher than master degree: 61.9%, bachelor degree: 38.1%), whereas Belgian employees were on average 35.3 years old (*SD* = 8.78), and also highly educated (higher than master degree: 75%, bachelor degree: 15%, secondary school or lower: 10%). Respondents’ tenure ranged from one to 35 years with the average being nine years six months for Chinese and eleven years three months for Belgian respondents. Respondents were active in some of the major sectors in both countries, including administration, engineering and manufacturing, finance, education, support services and government. Table 1 contains an overview of both samples’ demographic characteristics, as well as their scores on the measure of horizontal and vertical individualism/collectivism.

**Table 1:** Match of employee groups with Chinese and Belgian companies.

	Chinese	Belgian

Gender		
Male	8	10
Female	13	10
Sector		
Administration management	3	2
Engineering, manufacturing or production	4	3
Education and research	5	3
Finance or accounting	2	3
Government	4	7
Support services	3	2
Tenure		
More than 30 years	1	2
20–29 years	0	1
15–19 years	1	2
10–14 years	2	7
5–9 years	8	3
1–4 years	9	5
Education		
Higher than master	13	15
Bachelor	8	3
Secondary school or lower	0	2
Total	21	20
Horizontal/Vertical individualism/collectivism^a^		
Horizontal individualism	5.06	4.95
Horizontal collectivism	4.67	5.56
Vertical individualism	3.78	4.04
Vertical collectivism	4.80	4.01

^a^ Mean scores of both countries’ interviewees on the four dimensions according to the questionnaire results.

### Procedure

The interviews were semi-structured and conversational. We used seven questions to probe job background, reasons for choosing the current job and organization, and characteristics of the ideal job from respondents. These questions were meant to probe the core beliefs of PCs, and to gain insights into the features of the current and the desired exchange relationship: perceptions of obligations, promises, and general or normative expectations (Montes & Zweig, 2009; Rousseau et al., 2018). The interviews began with a few general questions on demographic and background information, allowing the interviewees to feel at ease (Parzefall & Coyle-Shapiro, 2011). The reasons for choosing their job and organization, as well as their ideal job, were probed because the features of the PC content could be inferred from each description. Respondents’ views of their ideal job were contrasted with perceptions of their actual job, giving us rich information on the general and normative expectations of their PC. Respondents were encouraged to provide as much detail as possible to increase the accuracy and quality of reported content.

We first interviewed Belgian employees, followed by the Chinese employees. Most of the interviews took place in homes or meeting rooms at the convenience of interviewees, while ensuring that the location was quiet to facilitate the interview. Belgian respondents were interviewed in English by the first author, whereas Chinese were interviewed in Mandarin, and then translated to English by the first author. We included English proficiency as an inclusion criterion when selecting Belgian interviewees. All interviews were audio-recorded and transcribed, and analysed by one author in an effort to minimize bias (Alvesson, 2003). Each interview lasted approximately thirty minutes to one hour, the average being 37.68 minutes, with an exception of one interview with a Belgian employee with the shortest tenure, which lasted only 15 minutes.

### Qualitative analytical approach

The data analysis procedure combines elements of grounded theory and of content analysis, in particular Template Analysis (TA) (Waring & Wainwright, 2008). In TA, text is analysed through the use of an analysis guide, or codebook, consisting of a number of categories or themes relevant to the research question(s). Researchers often start with some ‘a priori’ codes. Once ‘a priori’ themes are defined, the researcher begins reading the data. From these, a graphical template was constructed for both Belgian and Chinese participants. The template was based on the first interviews of both Chinese and Belgian employees and on the literature. The analysis was conducted in Nvivo 12 (Edhlund & McDougall, 2019).

To explore research questions, categories of PC types were pre-defined by the theory described previously (Hesse-Biber, 2010), and themes of more specific PC content were added as they emerged from the interview data. The interviews were read several times to obtain a sense of the whole. Then the text about the employee’s content of the PC was extracted and brought together into condensed meaning units and labelled with a code (Graneheim & Lundman, 2004). Recursive methods were used in the data analysis, with condensed meaning units being described by codes, which were then linked to sub-categories and categories. Finally, these categories are used to induce the themes that are used to address our research questions. The number of participants recruited for this study was based on the extent to which new themes and categories emerged from interviews, meaning that we stopped recruiting participants when saturation was reached, and no new categories emerged from the interview data. Using this approach 388 condensed meaning units were selected from the interviews and coded into categories and themes. Three independent raters achieved agreement in 95% of cases, and disagreements between raters were resolved via discussion.

## Findings

First, we tested whether there are differences between PC types (transactional, relational, balanced, and ideological) when comparing Belgian to Chinese employees. Each condensed meaning unit was assigned a PC type category. Belgian interviewees reported mostly balanced PC types (45.55%), with a relatively small proportion of transactional (21.47%), relational (28.8%), and ideological (4.19%) elements. Chinese interviewees reported mostly relational (46.7%) and transactional (47.21%) elements, with fewer balanced (6.09%) and no ideological (0%) elements in their PCs.

### Belgian interviewees’ PCs

We identified four themes and eight sub-themes (a list of themes and definitions with example quotes can be found in online Appendix 1). The four themes and their frequency of occurrence are: Job commitment & Work-life balance (37.99%), Stability & Development (31.29%), Support & Cooperation (16.13%), and Self-Direction & Belongingness (14.52%).

#### Job commitment & Work-life balance

The most commonly emerging theme captures the paradox between strong job commitment on the one hand and the importance of work-life balance on the other hand. The strong affective commitment to the job is apparent from the interviewees’ descriptions of their desire to put in a lot of effort as well as the enjoyment they derive from work. For example:

“I am always working at night and reading [for work] on the train, because I feel connected to my job.” (Belgian interviewee # 18)

This strong sense of commitment appeared to originate from the fit between the interests, values, and expertise of the employee and the content of the job itself. In other words, the sense of person-job fit led to interviewees caring deeply about their job, which in turn resulted in high work effort. It was also clear that interviewees chose jobs so that the job content matched their own interests, rather than working in an organization for reasons of status or prestige. For example:

“I love the planet, so that I want to make a difference. I like to work for a company that is trying to make the world better.” (Belgian interviewee # 9)

Although Belgian interviewees expressed a strong desire to dedicate a significant amount of time to work, they simultaneously stressed the importance of leisure time and the need to find an optimal balance between work and other life domains. It was clear from their answers that the importance of non-work domains did not diminish the importance of work itself. For example, one interviewee expressed a willingness to work extra, as long as the organization compensated this with extra holidays during the summertime.

“I decided to work hard at that time, and got extra holidays. So that I can use my holidays in summertime instead of [during regular] weeks.” (Belgian interviewee # 3)

In addition, Belgian interviewees stated that they tried to maintain some boundaries between work and non-work. Although the combination of strong job commitment and optimal work-life balance may appear somewhat paradoxical at first, interviewees explained that a healthy work-life balance was necessary to cope with stress from work. Several interviewees indicated that the ability to work from home also helped to obtain a better work-life balance.

“I try to keep a distance between work and private life. Sometimes it’s hard to work here, because you have to face the fact that people do really bad things, if you don’t understand [their] reasons you will be really shocked…So, [besides] your work, other good things could make you feel not everything is bad. I have family, friends, and hobbies that give me good feelings, so I can [cope] with the bad feelings at work.” (Belgian interviewee # 19)

#### Stability & Development

This theme pertained to the paradox between two sub-themes, namely a desire for stability versus a desire for development. Interviewees reported that they expected a stable work environment, both in terms of job security and job location. They explicitly sought employment contracts that offered long-term job security, for example to avoid stress and uncertainty associated with temporary employment contracts:

“I wanted a fixed position. I could not count on an unstable salary every month.” (Belgian interviewee # 9)

In terms of job location, interviewees looked for a job close to where they lived. This relates to stability, as the interviewees want to maintain a status quo in their life. In other words, interviewees did not want to relocate for their job, and they wanted an easy commute to their work.

“The job is not far away from my home so that I can take a train by 14 minutes, and then I can do a little walk.” (Belgian interviewee # 11)

In contrast with this desire for stability, interviewees expressed a need to develop themselves. They felt that it was important to continually learn on the job, either by following training courses or by gaining new experiences on the job itself. Lifelong learning was seen as a natural part of work. Moreover, interviewees wanted to be challenged at work, by applying their competencies and skills in challenging and creative tasks. Overall, interviewees appeared to be driven—in part—by a need for achievement. However, this need for achievement was not related to a career advancement goal. Instead, it seemed to originate from an innate desire for self-development.

“I have the obligation to study and develop myself. So I learn and improve myself on the job all the time.” (Belgian interviewee # 6)

#### Support & Cooperation

The third theme pertains to the obligation to receive support from the organization as an individual employee versus the obligation and desire to cooperate with others and reciprocate their positive treatments. In terms of support, interviewees indicated that they wanted to be recognized for their work, as exemplified by the quote below. Interviewees valued respect in the workplace and wanted to feel that the organization cared about them. In addition, interviewees felt that their organization ought to treat them fairly. They expected all employees to be treated in a just way.

“I think it’s very important that when you work and deliver some reports or outcome of your research; for me it’s really important that somebody really uses these outcomes to increase the performance of the organization rather than just say it’s a good research and put it away.” (Belgian interviewee # 18)

Linked to this support, interviewees indicated that they felt obligated to cooperate with team members and with other organizational actors. Adequate communication appeared to be a necessary condition to facilitate cooperation. An important recurring subtheme pertaining to cooperation and communication was equality between colleagues: several interviewees expressed that there were no hierarchical relations hampering communication and collaboration. A consequence of this equality was that they experienced greater responsibility to voice their own opinions and contribute to team processes.

“My previous company has a lot of communications; we have freedom to call everybody, every branch in the company, to collect the information. You have all the cards in your hands, then play it as you wish.” (Belgian interviewee # 15)

While interviewees desired equality at the workplace, they also perceived a need to cooperate to gain benefits for the collective group or organization. They were likely to use communication to get collective benefits in return for the efforts they devote to the job. One interviewee stated that communication is important in their daily activities because their previous efforts would be wasted without efficient communication. This indicates the presence of a norm of cooperation, meaning that parties work together for a common, mutual or underlying benefit.

“You might encounter a lot of mistakes of which you will be annoyed. At the moment you have to consult the figures with the manager or colleagues, then you will be inspired. Otherwise, you will lose a lot of time to correct all the issues in the finance field if you don’t have information or source…We have a lot of communications between each other [so] that everybody can get to do everything more efficiently.” (Belgian interviewee # 15)

#### Self-Direction & Belongingness

The last theme illustrates a paradox between the desire for autonomy and self-direction and the desire to belong to the organization. On the one hand, interviewees indicated that they wanted and received a great deal of autonomy in how they organize their work and in how they collaborate with others. Their organization offered them a lot of flexibility: many could decide on their own work hours and were able to work from home. In return, interviewees were flexible themselves, for example by compensating in some way (e.g., working during the evening) in return for stopping earlier at work.

“We have a lot of freedom; we can decide by ourselves…So I can decide who I really want to see, which file has to be done first.” (Belgian interviewee # 19)

On the other hand, interviewees expressed a strong desire to belong to a group. Although Belgian interviewees expressed that autonomy and flexibility were important in the employment relationship, identifying with their organization appeared to be a condition that facilitated self-direction. Interviewees stated that they viewed their organization as a family and had good personal relationships with colleagues and their supervisor. They identified with their organization also because they experienced a fit between their own values and those of the organization.

“When I saw this (the type of company) I thought it looks like something I want to do. Because the company deals with the (polluted) tracks on the roads, then they pay more attention on how to clear (the pollution of) the cars, for me that is important because I love the world that we live in. I think people shouldn’t be penalized of polluting by vehicles.” (Belgian interviewee # 9)

### Chinese interviewees’ PCs

Three themes and eight sub-themes emerged from the interview data (Online Appendix 2). These themes and their frequency of occurrence are: Steady security (70.27%), Hierarchy (24.99%), and Interpersonal relationships (7.4%).

#### Steady security

The most frequently emerging theme is steady security, which is composed of the sub-themes material and immaterial security, family security, and leisure security. Material and immaterial security pertained to a steady income, and interviewees often referred to expectations about income in view of their length of employment, job performance, domestic policies, and other benefits. For example:

“[As a civil servant] your final salary or bonus is not based on the content of your job, but on the length of employment and job title.” (Chinese interviewee # 2)

However, material and immaterial security was also strongly linked to status. Chinese employees work in either government-state or private owned corporations. The organization for which one worked was a strong determinant of (im)material security, with government departments or state-owned enterprises (SOEs) offering lower wages but more stability and other benefits such as housing funds. As one of the interviewees expressed, a stable job could make his life more secure, however, it was difficult to get a stable job within the competitive employment environment:

“I mainly want to find a job that is a bit more stable, because I am now older and can no longer go around like a young man, and I also want to start a family, but after I am married, I must have a good economic and work foundation…It is almost impossible to find a better institution in Liaoning Province with the current master’s degree level. So, I chose to take an exam in a provincial city, which was a relatively less competitive city.” (Chinese interviewee # 21)

Interviewees also sought security in view of their family life. In line with the traditional collective culture in China, interviewees cared deeply about their family, which resulted in high consideration of family factors when making work-related decisions. The interviewees were looking for a job which could improve their family life, such as founding a family and offering permanent residence permits (*Hukou*)^1^ (Démurger, Sachs, Woo, Bao, & Al, 2002). For example:

“When I was looking for work in Beijing, I must first solve the problem of the hukou system of household registration.” (Chinese interviewee # 9)

Finally, Chinese interviewees also wanted to secure opportunities for leisure activities. They viewed work as means to enable leisure activities outside of work and were willing to sacrifice pay to obtain more opportunities for leisure time by working in a government-state organization rather than in a private organization. For example:

“I did not know the content of this job before I was employed at the beginning; I just felt that it would be better to leave my current job at that time (because the content of that current job was too tiring for me). After I changed, I feel easier and [have more] leisure [time].” (Chinese interviewee # 6)

#### Hierarchy

This theme contains the sub-themes promotion, obedience to authority, and seniority, which align with Confucian tradition that offers considerable power depending on hierarchy (Hui et al., 2004). The promotion sub-theme captures interviewees’ aims to rise in the hierarchy and involved mostly explicit obligations and expectations about how and when promotions were offered. For example, one interviewee talked about the relationship between pay and advancement, emphasizing that rising in the hierarchy would lead to higher salaries, and explained that promotion depended on the length of employment but would ultimately be decided by the higher leadership.

“The only way to increase wages is getting a higher job title. To acquire a job title is divided into two parts. One is the evaluation system and the other is the employment system. You first need to have two years of qualified experience, and then you can apply for it. After application, it can be evaluated, but after the evaluation, it is not necessarily awarded (which means you may not get the corresponding salary even if you are promoted to the corresponding title). Evaluation and employment are separate.” (Chinese interviewee # 13)

A second sub-theme that emerged conveyed the belief that employees were obligated to obey their superiors. This aligns with the moral perspective on justice in which employees from high power distance cultures defer to power and are more likely to find obedience to power morally acceptable (Shao, Rupp, Skarlicki, & Jones, 2013). For example:

“When I graduated, I was directly assigned to this state-own enterprise. It was not my choice. It was the state distribution. In that era (the 80s), when I first entered the factory, I did not have the right to choose, but could only obey the assignment. In that era, once people graduated, the country allocated the package. After graduation, 20 of my classmates were all assigned to the current enterprise.” (Chinese interviewee # 20)

Hierarchy does not only shape the relationship between leaders and subordinates, but also between co-workers. Interviewees expressed that older employees possess more power and privileges in the organization, even though they are colleagues. For example, one interviewee explained that it is the culture of the company that newcomers work more than older employees. Likewise, the promotion system favoured older employees by emphasizing tenure as a key criterion for promotion.

“Because you are a newcomer, and you are young. You certainly have to do more than the older staff.” (Chinese interviewee # 1)

#### Interpersonal relationship

The last theme was clearly less prevalent in interviewees’ responses compared to the previous two themes. Interviewees reported that they expected a harmonious work atmosphere and that they wanted to improve their interpersonal skills, such as communication and cooperation skills. However, when looking at the three main themes, we can see that interpersonal relationships are intertwined in each theme. When talking about steady security, interviewees state that acquiring various securities depended on external conditions such as national policy, competition, and a high-pressure work-environment. They implicitly expressed that being social-savvy (Miller, 2010) was necessary in their current employment relationships, for example to gain better benefits or positions of security:

“I always feel that my job is stable and don’t want to change my job. Especially when I was a student, the school taught me very little about social reality… Just after graduating, I had to solve my problems of food, clothing, housing, and job…But now, looking at my personality and abilities; I may be more suitable to be a project manager. From the perspective of thinking, doing technology only concerned with what you do, you don’t have to care about what others want, but in the later period, I am willing to cooperate with others.” (Chinese interviewee # 7)

Interpersonal relationships were also intertwined with the hierarchy theme, ranging from relationships between supervisors and subordinates, and with coworkers. The high quality of these relationships was seen as an obligation for the interviewees, which led to a harmonious atmosphere between leaders and coworkers, as well as an advantage for survival in the employment relationships:

“No matter if this job is my responsibility. It is the work contents of the unit. If you do not do it, no one will do it. You cannot let the leader do it. So, I have to do it…To be a leader in our unit, in addition to having the ability; you have to look at the leader’s permission.” (Chinese interviewee # 1)

## Discussion

Our study explored cultural differences in PC content for Belgian and Chinese employees, interpreted through the lens of cultural values and contrasted against predominantly Western PC theory (Rousseau et al., 2018; Thomas et al., 2010). Our qualitative findings highlight differences in the content of Belgian and Chinese employees’ PCs. We found that Belgian interviewees perceived their PC as balanced (PCs based on compromises) and that these PCs reflect a horizontal collectivist culture. In contrast, Chinese interviewees perceived mixed contracts that primarily held relational content and secondarily transactional content (Utilitarianistic Guanxi PCs), and their PCs reflected a vertical collectivist culture. Four themes (Job commitment & Work-life balance, Stability & Development, Support & Cooperation, and Self-Direction & Belongingness), each with two sub-themes (Function & Role orientation and Work-leisure dichotomy; Fidelity and Self-Stimulation; Equity and Negotiation; Autonomy & Flexibility and Identity respectively), emerged from the Belgian interview data, with themes reflecting a balanced PC type. Three themes and eight sub-themes—Steady security (Material & immaterial security; Family security; Leisure security), Hierarchy (Promotion; Superior authority; Seniority), and Interpersonal relationships (Harmonious atmosphere; Interpersonal skill)—emerged from Chinese interview data; the first and second theme having both a transactional and a relational character, whereas the last theme had a relational character.

Our finding that Belgian interviewees’ PCs appear to be balanced fits a culture of horizontal collectivism which consists mainly of elements of equality, and balanced reciprocity. We found no strong evidence to show that Belgian employees’ PCs can be described using the PC typology of McLean Parks & Smith (1998) as either exploitive, instrumental, communitarian or custodial. The moderate climate seems to reflect Belgians’ attitude to life: the middle road between extremes. Therefore, we propose that a “culture of compromise” (Sels et al., 2000) best describes Belgian employees’ PCs. Our finding that Chinese interviewees’ PCs contained both relational and transactional elements and fit the cultural type of vertical collectivism is consistent with earlier studies claiming that relational contracts play a dominant role in Hong Kong (Kickul et al., 2004). Steady security was found to be the most important theme in our data, followed by hierarchy and interpersonal relationships. Exchange relationships appear to be based on the exchange of favours and controlled by strict hierarchical and social rules. This is consistent with Kickul and colleagues’ (2004) statement that Chinese society is “utilitarian and familism”. We therefore label the Chinese PC as ‘Utilitarianistic Guanxi’.

Zooming in on the themes and subthemes that emerged from the interviews offers a more nuanced understanding of Belgian and Chinese employees’ PCs. For Belgian interviewees, it was clear that they were seeking a balance between transactional and relational obligations, as well as between making contributions to the organization and receiving inducements in return. The latter search for balance highlights the quid-pro-quo nature of the exchange relationship that underlies the PC of Belgian interviewees (Rousseau, 1989; Rousseau et al., 2018). We captured this striving for balance by positioning themes as paradoxes that needed to be resolved. For example, Belgian interviewees were highly committed to their job, which translated into working hard and, sometimes, long hours. However, they also indicated that they desired a good work-life balance, with clear boundaries between work and other life domains. To resolve the tension between these two elements in the exchange relationship, they felt that the employer was obligated to give them autonomy and flexibility, so that they could decide by themselves when and how to organize work (e.g., by working from home). Likewise, they felt that their organization was obligated to offer opportunities for self-development—which fits with an individualistic mind-set—whereas they simultaneously expressed a desire to belong to a collective—which fits a collectivistic mind-set. This tension reflects the basic tenet of Optimal Distinctiveness Theory, which describes that people seek a balance between a need to be unique and a need to belong (Brewer, 2010). It also shows that—from a cultural values perspective—both individualistic and collectivistic values appeared important in Belgian interviewees’ PCs, albeit that collectivistic elements appeared to be slightly more dominant. More clearly, interviewees valued equality, which translated into PC obligations such as respect, an equal distribution of work and responsibilities, and having a voice in decision-making processes. From a cultural values perspective, this demonstrates the importance of a horizontal, rather than a vertical, value system (Thomas et al., 2010).

Similar to their Belgian counterparts, Chinese interviewees’ PCs contained both transactional and relational elements. However, they did not seek to balance both. Instead, their PC had a more layered structure with a strong transactional core (e.g., material and immaterial benefits). These transactional obligations served relational needs; for example, Chinese interviewees felt that their organization was obligated to offer them material benefits so that they would have the financial stability to start a family. This result is consistent with the findings of Kickul and colleagues (2004) in a Hong Kong based study that the primary responsibility of Chinese people is to provide economic resources or benefits for their families. Overall, these obligations in the PC align with utilitarian principles in Chinese society (Zheng, Wu, Chen, & Lin, 2017). A relational layer could be discerned on top of this transactional core in Chinese interviewees’ PCs, with relational elements appearing to be intertwined with all aspects of the exchange relationship. At work, this showed in the strong deference to authority that was expressed by Chinese interviewees. It was clear from the interviews that they experienced a strong obligation to obey their superiors. This tradition of hierarchy can be traced back to Confucianism and to a vertical cultural values system (Thomas et al., 2010). More broadly speaking, the importance of relationships in Chinese PCs can be linked to “guanxi”. Interpersonal relationships, or we could say ‘Guanxi’, play a key role in the employment relationship as it determines the benefits bestowed upon and the performance evaluation of employees (Wong, Wong, & Wong, 2010). Overall, ‘Guanxi’ can be considered to serve a utilitarian purpose. Therefore, we can describe Chinese PCs as ‘utilitarian guanxi’, which combines Chinese philosophy of pursuing profit and objective goods, such as material wealth, with family and social order (Fraser, Murphy & Bunting, 2005).

Differences in PC content between Belgian and Chinese interviewees could be attributed to various factors. One of the main forces underlying such differences could be cultural values. Belgian culture tends to be more moderate by keeping features of collectivism in balance with egalitarian interpersonal relationships of the horizontal dimension. In contrast, Chinese culture is based on Confucianism and Taoism (Fan, 2000) and emphasizes hierarchy and harmony in interpersonal relationships. It is a collectivistic-orientation combined with asymmetric power in interpersonal relationships. In addition, we try to acknowledge the cultural differences between mainland China and Hong Kong. Although both parts of China share cultural elements, such as Confucianism, Hong Kong employees are more concerned about economic gains benefitting the family than mainland Chinese employees (Kickul et al., 2004). In addition, Hong Kong has been influenced more by Western culture than mainland China, which may have stimulated self-development and a desire for being challenged. This influence of Western culture may thus have led to a divergence in values between Hong Kong and other areas in China (Ralston, Gustafson, Cheung, & Terpstra, 1993), resulting in different perspectives on employment relationships as well (Liu, Nauta, Yang, & Spector, 2017).

However, there are likely additional factors that drive differences in PC content between Belgian and Chinese employees. An alternative explanation for such differences is the labour market and economic system. Most European countries rely on formal institutions and systems, such as the European Union, for dealing with issues of employment. There are more similarities than differences between European countries, resulting in the organizational context having stronger effects than country or sector (Schalk & Soeters, 2008). This diversity in organizational contexts means that it is possible that the individualistic nature of Belgian employees’ exchange relationships offers more room to fit the job to the individuals’ personal values, leading to the creation of ideological elements in the PC. In contrast, an important element in Chinese interviewees’ PCs appeared to be the choice of organization for which they worked. Chinese interviewees chose to either pursue a position with potentially high financial rewards in private firms or a position with job security in state-owned organizations. At present, many Chinese employees, especially in inland provinces, prefer to work in state-owned organizations. Due to historical reasons, China had an administered labour system from the 1950s to 70s. During that period, there was limited mobility on the labour market, meaning that one’s first job was often one’s last, which some have described as the ‘the iron rice bowl’ system (Knight & Yueh, 2004). After the reform of 1978, the Chinese Communist Party created ‘preferential policies’, which allowed coastal Chinese provinces to integrate into the international economy (Démurger et al., 2002). However, these policies have disproportionally benefited coastal over inland provinces (Linge & Forbes, 1990) by giving them more job opportunities. For employees from inland provinces, ‘the iron rice bowl’ job is still the best option (Démurger et al., 2002). This is especially so for people from northern provinces—whom we focused on in this study. Chinese policies attempt to reduce the number of employees in state-owned firms, partly by marketizing public services. Most Chinese interviewees nonetheless expressed a desire to be employed in a stable position and organization, despite having a lower salary which may barely cover basic costs of life, rather than a high salary with low job security.

### Practical implications

We extend the literature on PCs by exploring cultural context as an important factor to understand the subtle nuances that can emerge in PCs (Coyle-Shapiro et al., 2019; Rousseau et al., 2018). We follow the suggestions by Coyle-Shapiro et al. (2019) that using a poly-contextual approach by including cultural differences is necessary to explain differences in employee behavior across countries. These cultural differences shape what employees deem important in exchange relationships and thus helps shape the content of their PC. This in turn may affect their day-to-day behaviors at work, as employees try to attain the inducements that match their goals (Chen et al., 2008).

This research has some important implications for managerial practice. First, this study stresses that differences in PC content exist between Belgian and Chinese employees. Such information can have important ramifications for managers of culturally diverse teams, or for Western companies that seek to extend their business activities to China (or vice-versa). For managers of Belgian employees, attention should be paid to offering autonomy and room for self-development, while simultaneously offering job stability and the possibility to belong to a team. In other words, managers of Belgian employees need to be aware that optimal management of their employees’ PCs requires seeking compromises. Turning to Chinese employees, our findings indicate that traditional Chinese culture has a strong influence and that employees pursue steady security, comply with a compliance to hierarchy, and establish their PCs around interpersonal relationships.

Second, this study stresses the importance for global organizations to pay attention to not only the PC of local employees of one country but also to the PC of expatriates with the host company. Organizations need to communicate clearly with their expatriates to make sense of the exchange terms in their dual PCs. Many expatriates will experience a cultural shock when they start working in a different culture. The host company can try to minimize incongruence in the interpretations of PC terms to avoid that expatriates perceive a breach of their PC. Our findings can offer a starting ground to discuss perceptions of obligations for Belgian and Chinese employees working in different cultural contexts.

### Limitations and future research directions

Our study has certain strengths and limitations. First, we started this study based on the PC literature—including the distinction between transactional, relational, balanced and ideological PC types—which originated from a Western perspective. Chinese interviewees can have different interpretations which may not exist in a Western perspective. We attempted to counter this bias by using independent raters with a combined Chinese and Belgian background. While this bias may still have affected our interpretation of the interviewees’ responses, we believe that the themes that emerged are apt thanks to the multicultural background of the authors of this study.

Second, our results may be influenced by selection bias, as we used convenience samples, meaning that our findings may not generalize to the entire Belgian or Chinese population. For example, most of the Belgian interviewees originated from the Flemish region. Further, interviews were conducted in English for Belgian interviewees and we included English proficiency as an inclusion criterion for our Belgian sample. However, this inclusion criterion likely led to an overrepresentation of highly educated employees in our sample, compared to the general Belgian population. Likewise, for the Chinese sample, we focused on employees working in mainland China, which is not representative for the entire Chinese population (Schalk & Soeters, 2008). That being said, the goal of our study was to explore differences in PC content rather than empirically examine whether such differences can be generalized to the broader population. For qualitative studies, such as ours, having a representative sample is not necessarily an end-goal. Future research could use quantitative techniques to assess the extent to which the identified differences in PC content generalize to the population.

Finally, we focused on understanding differences in PC content between Belgian and Chinese employees, using cultural values as a lens to interpret and explain these differences. Naturally, other contextual factors, such as economic conditions or political systems also play a role in the formation of employees’ PCs. We therefore encourage future studies to consider broadening these contextual factors beyond cultural values when studying cultural differences in PC content and processes.

Based on our findings, we offer two recommendations for future research. First, our study illustrates the presence of cultural differences in PCs, which can be explained based on the dimensions of vertical/horizontal collectivism versus individualism. Future research could expand on this by integrating other cultural values, such as power distance, and other societal or economic factors to study how PCs differ from country to country (Thomas et al., 2003). Second, we advise future studies to explore how employees’ evaluations of the psychological contract (i.e., breach and fulfilment) differ between cultures. Recent theoretical models explain how employees react in the aftermath of violation (Tomprou, Rousseau, & Hansen, 2015) and how their reactions may depend on the phase of their psychological contract (Rousseau et al., 2018). However, it appears important to study how culture shapes these effects.

## Additional File

The additional file for this article can be found as follows:

10.5334/pb.498.s1 Appendix. Explaination and quotes data related to the Interview.

## Footnotes

Hukou is a Chinese system in which the police issues residence permits to approved people. It restricts people to move within their cities and effectively denies the poor the possibility of getting a good job legally in prosperous cities. These migration controls are a major reason for the increasing inequality in the provinces of China.
