# Supplementary material for: Cultural Differences in The Content of Employees’ Psychological Contract: A Qualitative Study Comparing Belgium and China
Source: Psychol Belg. 2020 Jun 1;60(1):132–51. doi: 10.5334/pb.498 (PMC7274204; doi:10.5334/pb.498)
Supplement: Appendix. — Explaination and quotes data related to the Interview. [file pb-60-1-498-s1.pdf]

Appendix 1

Belgian PCs description and exemplary quotations

Description of culture and PC	Theme	Sub-theme	Definition	Example quotes
Horizontal Collectivist (Compromise culture PCs)	Job commitment & Work-life balance	Function & Role orientation	Based on employees' interests and job content instead of famous or infamous organization.	"*I am fascinated by the job which is about food production. People have not enough food in some parts of the world, while people eat too much in the other parts, much food is spoiled. And I think that's a way I got into Agriculture University. That's why I just started to develop myself and find out about things and what I was always interested in." (*#No. B8)<br><br>*"I love the planet, so that I want to make a difference. I like to work for a company that is trying to make the world better. But the thing is in this city, there are few jobs, not many opportunities for me to do this ideal job." (*#No. B9)
		Work-leisure dichotomy	Employees intent to do a good job in terms of quality and quantity that frequently overtime the contracted hour, which also intent to be compensated by related holidays. However, work and life should be separated that to be balanced.	*"I try to keep a distance between work and private life. I still like my job, sometimes it's hard to work here, because sometimes you have to face the fact that people do really bad things, if you don't understand the reason and you will be really shocked, then you are getting to think of the world is changed. So, behind your work, other good things that make you feel not everything is bad. I have other things to do, I have family, friends, hobby, that gives me good feelings, so I can go to work with the bad feelings. Sometimes it was hard to find the balance, but now I found it."* (#No. B19)
	Stability &	Fidelity	A devotion and faithfulness	*"I want a fixed position. It could not count on an unstable salary every month. I saw this*

Development		action fundamentally to the job or organizations.	*was an opportunity to get a stable job because I think that's really important." (*#No. B9)<br><br>*"But I was more interested in Bruges, because it was close to my hometown then where I lived at home with my parents." (*#No. B13)
	Self-Stimulation	A self-guided improvement (APA Dictionary of Physiology, 2007) through various ways that makes a person constantly excited or challenge in life.	*"I chose this job because it's so exciting when I heard about this at first. Because it's not only about the organization itself, it also has a lot responsibilities, because the previous company was very monotonous. It was repetitive every day. Now my job is very challenging in every aspect."(*#No. B4)
Support & Cooperation	Equity	Try to understand and give employees what they need to enjoy full, healthy lives; it is equal to 'more for those who need it'.	"*My leader is very social and I like him because his beliefs are that when there are a lot of external pressures you need to lower it internally. So he is not forcing people, he just let everybody what they want on their own rhythm.*" *(*#No. B8)
	Negotiation	Resolve points of difference, to gain an advantage for an individual or collective, or to craft outcomes to satisfy various interests. It is often conducted by putting forward a position and making small concessions to achieve an agreement.	"*If I do not agree with some points, I can express it. Because maybe it's not a good interpretation from my perspective, or their side. We have to discuss it." (*#No. B15)
Self-Direction & Belongingness	Autonomy & Flexibility	Job tasks or working hours can be decided by the employee themselves, while allowing time off to deal with personal needs.	"*We can decide to work between 7:30 to 9:00 and you have to work 8 hours, so if you start early you can stop early…You have a lot of freedom too, we can decide by ourselves, we have some reports that have to be done there, but how many conversations with this person,*

			or how you write something which is our freedom. So I can decide who I really want to see, which file has to be done at first, so that's also a good thing." (#No. B19)
	Identity	A feeling of belonging to a group. It is part of a person's self-conception and self-perception, which is related to the organization or group that has its own distinct boundary.	"*We are a little and small group and we are in fact like a little family. We are all together in five days of seven hours. And tomorrow we have a team building, we do walk in Brussels." (*#No. B10)

Appendix 2

Chinese PCs description and exemplary quotations

Description of culture and PC	Theme	Sub-theme	Definition	Example quotes
Vertical Collectivist (Utilitarian Guanxi culture PCs)	Steady security	Material & immaterial security	(1) Income that depends on the length of employment, performance, domestic policy, workload, and benefits.	"*My final salary or bonus is depending on the years of employment or job titles. It is not decided by my job contents.*" (#No.C2) "*My company pays five insurances and housing provident fund in line with national standards.*" (#No.C15)
			(2) Enter into either government-state includes state-owned enterprise, civil services system, and government-affiliated institutions that means permanent position or power or non-state-owned firms with high income relatively.	*"In China, the steadiest job is in the civil service system. No matter how much money you earn, we only want the job is stable."* (#No.C1) *"Due to the previous experience that many leaders start from this position, it should have prospects for development in my opinion."* (#No.C5)
		Family security	The organization's location close to home, work, and live with partners in the same city, and work for the family instead of self.	"*No matter working in any cities or any jobs, it depends on my partner.*" (#No.C8)
		Leisure security	Both the explicit relax time or space and implicit	"*It has summer and winter vacations, and I basically have more than 4 or 5 hours of leisure time during the 7 work hours so that I can do my own*

		mental free time or situation.	*things."* (#No.C8)
Hierarchy	Promotion	The advancement of an employee's power rank or position.	"*In public institutions, the year is the only way to obtain technical titles. The first five years of the work is a starting point of evaluating."* (#No.C13)
	Superior authority	The top leader has the absolute authority to all the decisions. Employees have to obey the leaders and follow their expectation to obtain the leaders' preference and approval.	"*To get a promotion in our organization, which is not a competition or selection, depends on leaders."* (#No.C6).
	Seniority	A person or group of people, who is older or has occupied a particular position longer, take precedence resource, and control power.	"*Newcomers have to do more than the old staff certainly."* (#No.C1)
Interpersonal relationship	Harmonious atmosphere	It refers to the extent to which individual values peaceful and concordant interpersonal relationships (Oyserman, Coon, & Kemmelmeier, 2002), including teamwork, benevolence to each other.	"*I wish my working environment is harmonious without intrigue. All the colleagues focus on how to do the work well rather than having their own ideas."* (#No.C12) "*The company's culture is that leaders make a private communication with employee accompanied by sympathy and solicitude."* (#No.C5)

	Interpersonal skill	Persuasion, listening, delegation, coordination, negotiation, social perceptiveness, and stewardship skill.	“*As an employee, I have to learn the skill of how to communicate and how to do things successfully with people.*” (#No.C8)
